# Novel sulphamoylated 2-methoxy estradiol derivatives inhibit breast cancer migration by disrupting microtubule turnover and organization

**DOI:** 10.1186/s12935-018-0719-4

**Published:** 2019-01-03

**Authors:** Rustelle Janse van Vuuren, Mandie Botes, Tamarin Jurgens, Anna Margaretha Joubert, Iman van den Bout

**Affiliations:** 1 Department of Physiology, University of Pretoria, Pretoria, 0084 South Africa; 2 Centre for Neuroendocrinology, University of Pretoria, Pretoria, 0084 South Africa

**Keywords:** Breast cancer, 2-Methoxyestradiol, Migration, Microtubules, Focal adhesion

## Abstract

**Background:**

The estrogen metabolite 2-methoxyestradiol (2ME2) and a number of synthesised derivatives have been shown to bind to microtubules thereby arresting cancer cells in mitosis which leads to apoptosis. In interphase cells, microtubules play an important role in the delivery of proteins to subcellular locations including the focal adhesions. In fact, focal adhesion dynamics and cell migration are in part regulated by microtubules. We hypothesised that novel 2ME2 derivatives can alter cell migration by influencing microtubule dynamics in interphase cells. In this report we describe 2ME2 derivatives that display anti-migratory capabilities in a metastatic breast cancer cell line through their effects on the microtubule network resulting in altered focal adhesion signalling and RhoA activity.

**Methods:**

Cell migration was assayed using wound healing assays. To eliminate mitosis blockage and cell rounding as a confounding factor cell migration was also assessed in interphase blocked cells. Fluorescence confocal microscopy was used to visualise microtubule dynamics and actin cytoskeleton organisation while western blot analysis was performed to analyse focal adhesion signalling and RhoA activation.

**Results:**

2ME2 derivatives, ESE-one and ESE-15-one, inhibited cell migration in cycling cells as expected but equally diminished migration in cells blocked in interphase. While no significant effects were observed on the actin cytoskeleton, focal adhesion kinase activity was increased while RhoA GTPase activity was inhibited after exposure to either compound. Microtubule stability was increased as evidenced by the increased length and number of detyrosinated microtubules while at the same time clear disorganisation of the normal radial microtubule organisation was observed including multiple foci.

**Conclusions:**

ESE-15-one and ESE-one are potent migration inhibitors of metastatic breast cancer cells. This ability is coupled to alterations in focal adhesion signalling but more importantly is associated with severe disorganisation of microtubule dynamics and polarity. Therefore, these compounds may offer potential as anti-metastatic therapies.

## Background

According to the National Cancer Registry of South Africa, 1 in 27 South African women will get breast cancer during their lifetime [1]. Breast cancer metastasis is the foremost cause of mortality among breast cancer patients due to the impact the secondary tumours have on essential organs. Successful metastasis depends on a complex sequence of events that allow epithelial cancer cells to alter their adhesion and migratory behaviour leading to invasion of the surrounding stroma. This initial step is followed by intravasation of local vasculature, dispersion to distant sites, extravasation from the vasculature, and colonisation and growth within a distant environment to form a metastatic tumour (reviewed in [2]). One of the essential abilities of such metastatic cells is their ability to adhere to and migrate on extracellular matrix (ECM) proteins foreign to normal epithelial cells such as collagen (reviewed in [3]). It follows that inhibition of cell migration is a worthwhile target for therapeutic intervention since this will prevent invasion and metastasis and result in diminished mortality amongst patients.

Cell migration is promoted through actin branching at the leading edge of the cell leading to lamellipodia formation that allows cells to extend their cell membrane and adhere to new parts of the ECM. Adhesion to the ECM is dictated by the focal adhesions which form around clustered, engaged integrin dimers that are linked to the actin cytoskeleton via linker proteins including vinculin, talin and paxillin. Ligand bound integrins also perform cell signalling duties through binding proteins such as focal adhesion kinase (FAK) [4] and Src [5]. In fact, FAK phosphorylation at Y397 is directly correlated to the clustering and activation of integrins within focal adhesions [4]. Src and FAK activate pathways leading to cell survival, actin fibre organisation and proliferation (reviewed in [6]).

Microtubules are involved in the organisation and turnover of focal adhesions (reviewed in [7, 8]). This was first shown using fluorescently labelled tubulin and vinculin which extensively co-localised at cell adhesion sites [9]. This interaction of a microtubule with a focal adhesion is associated with a pause in microtubule growth followed by a rapid depolymerisation of the microtubule [10, 11]. Upon repeated targeting by microtubules, disassembly of focal adhesions is initiated which correlates with reduced FAK Y397 phosphorylation [9, 11].

Estrogen metabolises to form the intermediate metabolite 2-methoxyestradiol (2ME2). 2ME2 has both antimitotic and antiangiogenic activity in vitro suggesting its potential as an anti-cancer compound [12, 13]. Its mode of action has been shown to be through impacting on microtubule dynamics by interacting with the colchicine binding domain on β-tubulin [13, 14]. This leads to the abrogation of spindle fibre formation during mitosis, arrest of cells at the G_2/M_ border of the cell cycle and eventual apoptosis [15, 16]. In vivo, the 17-hydroxy group of 2ME2 makes it a target for 17-hydroxysteroid dehydrogenase-mediated metabolism in the gastrointestinal tract (GIT) and liver resulting in rapid metabolism [17]. Analysis of 2ME2 in phase I clinical trials showed rapid clearing of the compound [18].

To increase the effectiveness of 2ME2 and to reduce its metabolism and clearance from the blood, several derivatives have been designed. Previously, several sulphamoylated derivatives were designed including 2-ethyl-3-*O*-sulphamoyl-estra-1,3,5(10),15-tetraen-3-ol-17-one (ESE-15-one) and 2-ethyl-13-methyl-17-oxo-7,8,9,11,12,13,14,15,16,17-decahydro-6-cyclopenta[*a*]phenanthrane-3 sulphamate (ESE-one) [19]. The sulphamoyl group was added to increase the affinity of the compounds for carbonic anhydrase IX (CAIX), which is expressed on the cell surface of red blood cells. By allowing binding to CAIX it was envisaged that the compounds would bypass the first pass metabolism in the liver. Both ESE-15-one and ESE-one have been shown to be effective anti-proliferative agents in a number of cancer cell lines in vitro being effective at nanomolar concentrations [20]. They were shown to induce cell death through arresting the cell cycle and inducing apoptosis [19, 20]. In vitro, these compounds bind to and affect microtubule formation although no data has been published regarding their ability to inhibit microtubule formation in cells [21].

In this study, the anti-migratory potential of ESE-15-one and ESE-one was assessed as a measure of their potential use as anti-metastatic compounds. Standard wound healing assays were used, but the methodology was adapted to remove the confounding effects of cell proliferation and circumvent the compound’s ability to arrest cells at the G_2/m_ border which leaves cells rounded and non-migratory. In these interphase cells microtubule dynamics, focal adhesion signalling and Rho GTPase activity were analysed. The data shows that the compounds inhibit interphase cell migration. This is associated with a loss of microtubule organisation and polarity as well as increased tubule stabilisation. Furthermore, focal adhesion kinase signalling was increased while RhoA GTPase activity was inhibited. Together, these suggest that the compounds may inhibit migration by impacting on the microtubule dependent cell polarity and focal adhesion turnover.

## Materials and methods

### Reagents

#### Cell culture

MDA-MD-231 cells were cultured in DMEM medium containing 10% heat-inactivated foetal calf serum (FCS), penicillin G, streptomycin and fungizone. To exclude effects of serum induced proliferation on cell migration and to avoid the known effect of the tested compounds on mitotic arrest which leads to cell rounding that would act as a confounding factor in migration assays, cells were blocked in the G1/S phase (interphase) of the cell cycle. To arrest cells in interphase, they were incubated for 18 h in medium containing excess thymidine (2 mM) which inhibits DNA synthesis thereby activating the checkpoints which stop cells proceeding to mitosis.

#### Cell survival assay

Crystal violet staining was used to quantify cell numbers and in turn cell viability after exposure to ESE-15-one and ESE-one. MDA-MB-231 cells (5 × 10^3^) were seeded in 96 well tissue culture plates with 200 µl medium per well and allowed to attach overnight. After attachment, cells were exposed to DMSO (v/v %), ESE-15-one or ESE-one at a concentration series of 0.05 µM, 0.1 µM, 0.2 µM, 0.5 µM and 1 µM for 24, 48 or 72 h. At termination cells were fixed with 1% gluteraldehyde in distilled water for 15 min at room temperature (RT). The gluteraldehyde solution was discarded and crystal violet stain in distilled water was added (1 g/l) per well. Cells were incubated for 30 min at RT. After incubation the stain solution was discarded, and plates submerged and washed in tap water to remove excess stain. Plates were left to dry overnight. The dye was solubilized in dye 0.2% Triton X-100 in distilled water. The absorbency was read using an ELx800 Universal Microplate Reader spectrophotometer (Bio-Tek instruments Inc, Vermont, USA) at a wavelength of 570 nm. Each experiment consisted of six technical repeats and three biologically independent repeats were performed. Graphs represent the average of these experiments with error bars representing the standard error of the mean. To determine statistical significance a student’s *t* test was performed.

#### Migration assay

The effects of the compounds on cell migration was determined by growing MDA-MB-231 cells to confluency, pre-treating cells and performing a monolayer scratch assay. Cells were confluent after seeding 1.75 × 10^5^ in 24 well plates and allowing attachment overnight. Cells were scratched vertically and horizontally using a pipette tip. The area of removed cells allowed the cells along the edges of this area to migrate into the generated space. Measurement of the closure of this space after 18 h is directly related to the efficiency of migration after exposure to the compounds. Assays were performed for both cycling cells and cells blocked in interphase. Images were taken on a Zeiss Inverse Axiovert CFL40 microscope (Carl Zeiss, Goettingen, Germany) using a 4× magnification objective and the scratched area was photographed and marked. Images were analysed using ImageJ software. Three technical repeats were performed per well with a minimum of 4 wells per condition for each experiment. At least three biologically independent repeats were performed.

#### Confocal imaging

Confocal microscopy was used to visualise actin fibers, nuclei and microtubules. Cells were plated in 24 well plates (5 × 10^4^/well) with each well containing a sterilised coverslip. After overnight incubation to allow attachment, cells were treated with the compounds and DMSO as vehicle control for 18 h.

##### Staining of actin cytoskeleton

At the end of the time point cells were fixed by incubation with a 2% paraformaldehyde solution for 15 min at RT. Wells were washed thrice with PBS before cells were permeabilised using a 0.2% triton X-100 solution for 5 min at RT. Cells were washed thrice in PBS and incubated with blocking solution containing 2% BSA in PBS for 60 min. Next, cells were incubated with blocking solution containing a 1:500 dilution of phalloidin conjugated to dsRED along with 1 μg/ml DAPI as a DNA counterstain for 1 h, RT.

##### Staining of stable and dynamic microtubules

To assess the effect of ESE-15-one and EE-15-one on microtubule stability, stable and dynamic microtubules were visualised using antibodies directed at tyrosinated and detyrosinated tubulin (Kind gift from Laurence Lafanechère). Cells were plated on sterile coverslips and blocked in G_1/S_ by incubation with thymidine for 18 h before they were exposed to the compounds. At termination cells were fixed using ice cold methanol for 10 min and washed three times in PBS. Cells were permeabilised in PBS containing 0.2% triton X-100 for 5 min, washed three times in PBS and blocked in PBS containing 2% BSA for an hour at RT. A dilution of 1:4000 mouse anti-tyrosinated α-tubulin Ab and 1:4000 rabbit anti-detyrosinated tubulin Ab was added to cells and incubated for 1 h at RT. Cells were washed in PBS and incubated with anti-mouse and anti-rabbit FITC Ab, respectively for 1 h at RT.

Wells were washed thrice and mounted. Slides were left overnight at room temperature to allow mounting fluid to harden before being imaged with a Zeiss 510 META confocal laser microscope. Images were analysed using ImageJ software.

### Western blotting

FAK phosphorylation was assessed by western blotting. Cells were plated at 2 × 10^5^ cells/well in 6-well culture plates and grown overnight before they were exposed to ESE-15-one or ESE-one. At termination, dishes were washed twice in cold PBS before cells were lysed. Cells were lysed in 0.5 ml of lysis buffer on ice and collected by scraping. Lysates were cleared by centrifugation for 5 min at 4 °C. Protein samples were prepared for resolution on SDS-PAGE gel with 4× LDS buffer and 2.5% β-mercapto-ethanol, heated, centrifuged and loaded onto the gel. Gels were resolved in MOPS running buffer under 120 volts for an hour. To determine protein sizes, a protein size marker, SeeBlue^®^ Pre-Stained Standard was loaded in one lane. Membranes were blocked by incubation in PBS-T containing 2% milk powder and rinsed in PBS-T before incubation with anti-FAK and anti-phospho-FAK antibodies for an hour. Membranes were washed with PBS-T and incubated with anti-rabbit IgG antibody conjugated to horse radish peroxidase (HRP) for 1 h. Membranes were washed and incubated with ECL substrate for 2 min before being imaged using a BioRad GelDoc system 1 (Bio-Rad Laboratories). Using Image Lab™ software 5.2.1 (Bio-Rad Laboratories) band intensities were quantified. Experiments were repeated 3 times and the relative band intensity averaged. To determine statistical significance a student’s *t*-test was performed.

### Rho GTPase activity assay

Cells were seeded in sterile petri dishes (5 × 10^6^) for RhoA detection. Cells were grown overnight before being blocked in the G1/S phase with the addition of thymidine (2 mM) for 18 h and exposed to DMSO as vehicle control, EE-15-one and ESE-one for 18 h. At termination dishes were washed twice in cold PBS before cells were lysed. Cells were lysed in 0.5 ml of lysis buffer on ice and collected by scraping. Lysates were cleared by centrifugation at for 5 min at 4 °C. Cleared lysates were incubated with Rhotekin beads for 1 h at 4 °C with continuous rotation. Afterwards, beads were collected by centrifugation for 20 s, washed three times in lysis buffer and resuspended in LDS loading buffer. Beads were incubated at 80 °C for 10 min before supernatant was loaded on SDS-PAGE gel and transferred to membrane. Membranes were probed for RhoA proteins using the mouse anti-RhoA antibody (Sigma Aldrich, St. Louis, MO, USA).

## Results

### ESE-15-one and ESE-one reduce cell proliferation

**Fig. 1:**
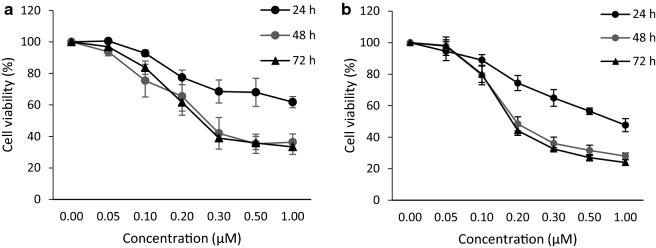
ESE-15-one and ESE-one reduce proliferation of MDA-MB-231 cells. MDA-MB-231 cells were exposed to increasing concentrations of ESE-15-one (**a**) of ESE-one (**b**) for 24 h (filled black circle), 48 h (filled grey circle) or 72 h (filled triangle). Cell survival was measured by crystal violet staining and quantification. Both experiments were repeated three times and each graph represents the average with the error bars representing the standard error of the means (SEM)

ESE-one and ESE-15-one have previously been shown to inhibit mitosis in different cancer cell lines [20]. Dose response curves were performed for both compounds in MDA-MB-231 cells at 24, 48 and 72 h post exposure. ESE-one and ESE-15-one both reduced cell survival at nanomolar concentrations with respective maximum effects reached at 48 h (Fig. 1). ESE-one is slightly more effective with a maximal reduction in cell number of 76.1% while ESE-15-one reduced cell number by 66.6%. These data suggest that the compounds inhibit proliferation after 48 h exposure while a significant number of cells remain present even after 72 h at concentrations of 0.5 μM or more.

### ESE-15-one and ESE-one inhibit cell migration independently of their effect on cell division

**Fig. 2:**
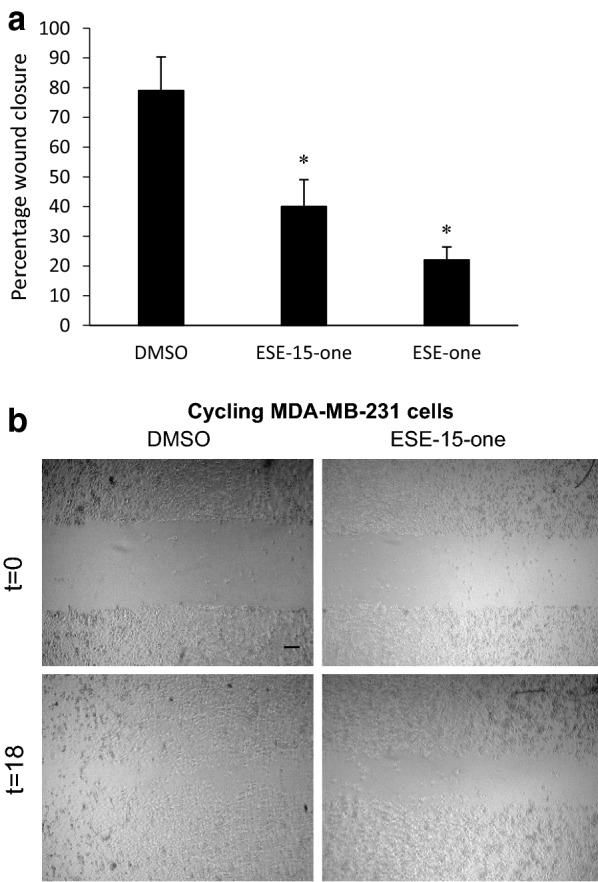
ESE-15-one and ESE-one significantly inhibit MDA-MB-231 cell migration. **a** Cell migration was quantified using a wound closure assay where monolayers of MDA-MB-231 cells were exposed to 0.2% DMSO as vehicle control, or to 0.5 μM ESE-15-one or 0.5 μM ESE-one directly after a cell-free zone was generated. Cell migration into the cell-free zone was quantified after 18 h. An average of three independent experiments was calculated with error bars representing SEM, while *P < 0.001 in a t-test between vehicle control and each compound respectively. **b** Light microscopy images of cell migration assays showing cycling cells at time 0 h and after 18 h treated with DMSO or ESE-15-one. Scale bar is 200 μm

**Fig. 3:**
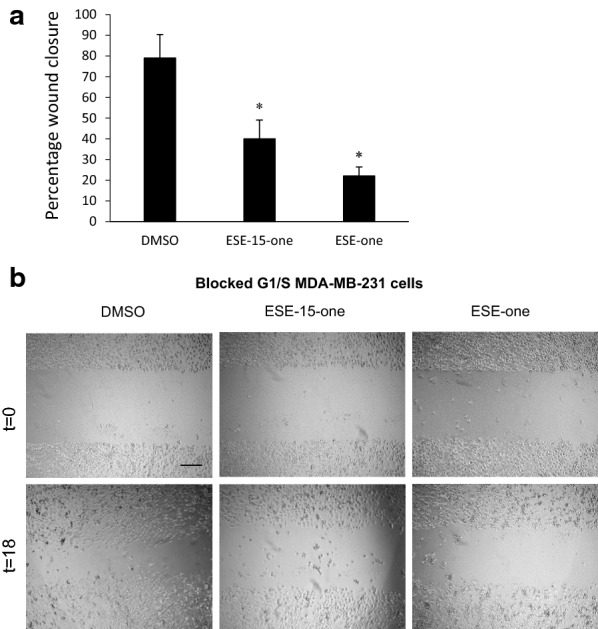
Migration of interphase arrested cells is inhibited by ESE-15-one and ESE-one exposure. **a** MDA-MB-231 cells were first blocked in G_1/S_ by exposure to thymidine before cell free zones were generated and cells were exposed to 0.2% DMSO, 0.5 μM ESE-15-one or 0.5 μM ESE-one. Cell migration into the cell-free zone was quantified after 18 h. The graph represents the average of at least three repeats with error bars representing SEM. *P < 0.001 in t-test comparison with DMSO-treated cells. **b** Light microscopy images of cell migration assays showing interphase cells at time 0 h and after 18 h treated with DMSO, ESE-15-one or ESE-one. Scale bar is 400 μm

While it is known that 2-methoxyestradiol (2ME2) and its derivatives are potent antimitotic compounds, their ability to inhibit breast cancer cell migration has not been studied. To analyse the effect of the 2ME2 derivatives ESE-one and ESE-15-one on cell migration, wound closure assays were employed using MDA-MB-231 cells. Cells were plated and allowed to reach confluence before a cell-free zone was generated after which DMSO, 0.5 μM ESE-one or 0.5 μM ESE-15-one was added. The cell-free zone was measured at 0 h and 18 h (Fig. 2). In control cells, the wound closed by 79% after 18 h. In contrast, cell migration was significantly inhibited after exposure to ESE-15-one and ESE-one with the wound closing by only 40% and 22%, respectively. This data suggests that these compounds have potent anti-migratory capabilities. However, disruptive effect of these compounds on cell division may act as a strong confounding factor since exposed cells arrest at the G_2/M_ border of the cell cycle leaving cells rounded and non-migratory [20]. To exclude the effect of cell rounding on cell migration, cells were first arrested in interphase by treating them with high concentrations of thymidine. Thymidine exposure saturates the replication machinery resulting in the blockage of DNA replication and subsequent arrest of cells in the G_1/S_ phase or interphase [22]. Cells blocked in this phase are spread and remain migratory. Thymidine treated cells were plated for migration assays and exposed to ESE-15-one, ESE-one or dimethyl sulfoxide (DMSO) immediately after a cell-free zone was established (Fig. 3). Interphase arrested cells treated with vehicle control migrated less than cycling cells (79% vs. 50% wound closure) suggesting that the arrest of cells in interphase partially inhibits migration which is an indication of the confounding influence of cell proliferation. Nonetheless, both ESE-15-one and ESE-one exposure still resulted in substantial reductions in wound closure (20% and 12% respectively).

Thus, a similar relative reduction in cell migration is measured in cycling and non-cycling interphase cells exposed to ESE-15-one (39% reduction in cycling cells vs. 30% in interphase cells) or ESE-one (57% reduction in cycling cells vs. 38% reduction in interphase cells). Overall, these data suggest that ESE-15-one and ESE-one are potent inhibitors of metastatic breast cancer cell migration independently of their antimitotic activity.

### ESE-15-one and ESE-one interfere with microtubule dynamics and organisation but do not affect actin cytoskeleton organisation

**Fig. 4:**
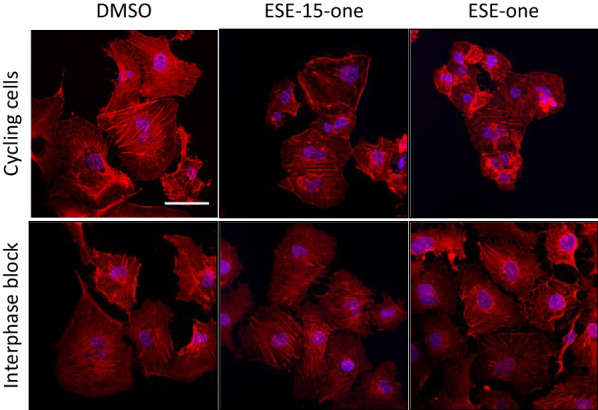
ESE-15-one and ESE-one exposure does not affect actin cytoskeletal organization but does affect nuclei morphology. Cycling cells or interphase arrested cells were exposed to 0.5 μM ESE-15-one or 0.5 μM ESE-one for 18 h before being fixed and stained for F-actin (red) and nuclei (blue). DMSO was used as vehicle control. Images were obtained on a LSM510 with a ×63 mag objective. Scale bar = 20 μm

Cell migration is dependent on a functioning and properly organised actin cytoskeleton. We hypothesised that the inhibition of cell migration occurs as a result of alterations in actin cytoskeleton organisation or formation. To test this, cycling cells or interphase arrested cells were exposed to 0.5 μM ESE-15-one or 0.5 μM ESE-one for 18 h, fixed, and stained for actin fibres and nuclei using phalloidin and DAPI respectively (Fig. 4). In control cells, some stress fibres were present throughout the cells with additional fibres along the cell periphery. Cells exposed to ESE-one or ESE-15-one did not display any visible changes from the control cells and had a similar actin architecture with no visible alterations in density or organisation of the fibres. In contrast (4′,6-diamidino-2-phenylindole) DAPI staining in cycling cells revealed that nuclei morphology was affected after exposure to ESE-15-one or ESE-one with exposed cells containing multiple nuclei or micronuclei in contrast to single nuclei in control cells. This effect was, however, mostly limited to cycling cells with only the occasional interphase blocked cell exposed to either compound presenting multiple nuclei. These data suggest that the compounds do not have visible effects on the actin cytoskeleton but their antimitotic effect on nuclei morphology is visible in cycling cells. Therefore, blocking cells in interphase removes the antimitotic effect of the compounds tested.

**Fig. 5:**
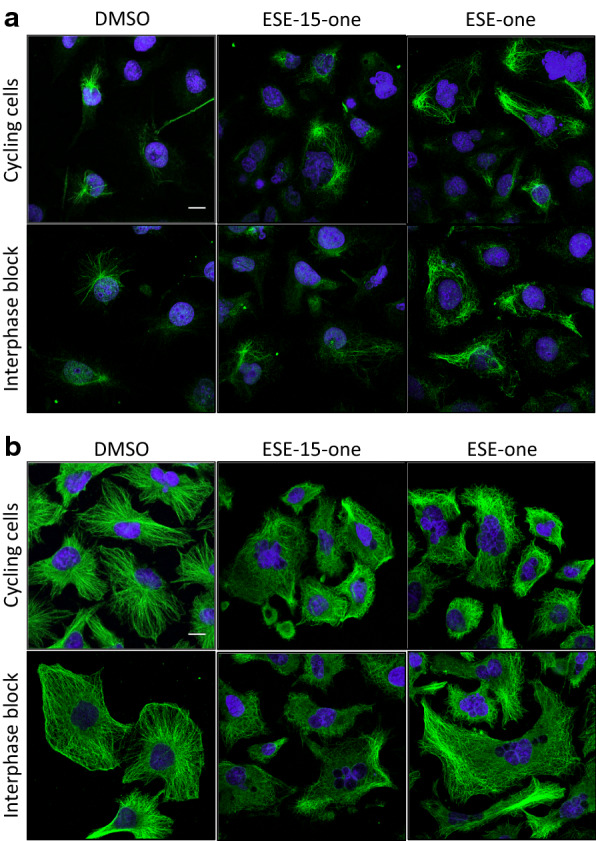
ESE-15-one and ESE-one exposure stabilize and disorganize microtubules. **a** MDA-MB-231 cells were either cycling or arrested in interphase and treated with DMSO, ESE-15-one or ESE-one for 18 h after which detyrosinated, stabilized microtubules (**a**), or tyrosinated, dynamic microtubules (**b**) (green) were visualized along with cell nuclei (blue). Images were obtained on a LSM510 with a ×63 mag objective. Scale bar = 5 μm

Microtubules target focal adhesions repeatedly leading to their disassembly and turnover. This process is important during migration when continuous formation and disassembly of focal adhesions are needed for persistent migration. 2ME2 and the sulphamoylated derivatives have been shown to bind to microtubules and affect their turnover in vitro. We hypothesise that ESE-15-one and ESE-one could affect cell migration by influencing microtubule dynamics. In normal cells, microtubules tend to radiate from the centrosome to the periphery with stabilised microtubules mainly found around the centrosome and dynamic microtubules extending into the cell periphery. In migrating cells, the centrosome is mostly localised between the nucleus and the leading edge. Cycling or interphase arrested MDA-MB-231 cells were exposed to 0.5 μM ESE-one or 0.5 μM ESE-15-one for 18 h and processed. Stabilised microtubules were visualised using an antibody directed against detyrosinated tubulin while dynamic microtubules were visualised using an antibody directed against tyrosinated tubulin. DMSO exposed cells revealed a single, radiating array of stabilised microtubules that extended only a short distance from the centrosome (Fig. 5a) with no obvious differences between cycling and interphase arrested cells. In contrast, cells exposed to ESE-15-one or ESE-one contained increased numbers of stabilised microtubules with many extending well into the periphery of the cells. Moreover, in many cells the single point of origin for microtubules was lost and stabilised microtubules radiated from multiple points throughout the cell or were present in parallel bundles along the periphery of the cell. This phenomenon was not dependent of cell cycle progression as it was visible in interphase arrested cells too. Similar treated cells processed for dynamic tubules with the anti-tyrosinated tubulin antibody displayed dynamic microtubules throughout the control cells with a strong radial pattern extending into the cell periphery and no visible differences between cycling and interphase arrested cells (Fig. 5b). However, exposure to ESE-one or ESE-15-one resulted in gross disorganisation of this radial pattern. Moreover, multiple points of origin were present in some cells while others completely lacked such foci. Many microtubules also appeared to extend underneath and in parallel to the cell membrane. These data suggest that the microtubules are disturbed in such a way that targeting and delivery of biomolecules to focal adhesions would be impaired. This phenomenon has also been associated with a loss in cell polarity and resulting decreased migration.

### Microtubule stabilisation and decreased cell migration after ESE-15-one and ESE-one exposure is associated with altered focal adhesion signalling and RhoGTPase activity

**Fig. 6:**
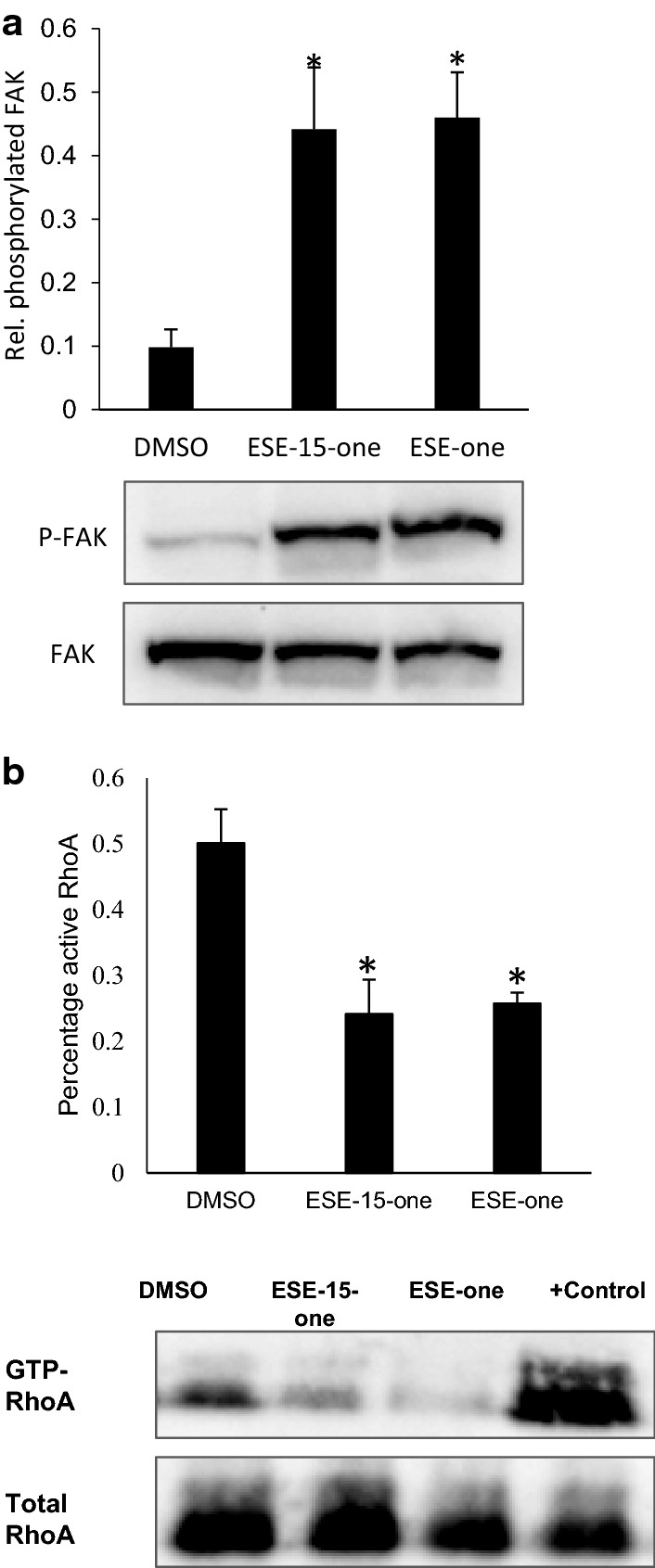
ESE-15-one and ESE-one increase FAK phosphorylation in G1/S arrested MDA-MB-231 cells. **a** Western blot analysis of interphase arrested cells exposed to ESE-15-one or ESE-one was performed with antibodies directed against phosphorylated Y397 FAK or total FAK. The average of 3 experiments were quantified using densitometry and relative FAK phosphorylation was calculated by dividing the phosphorylated FAK levels by the total FAK levels. The graph shows the average with error bars representing SD. *P-value < 0.05 when compared to the DMSO control. **b** Peptide pulldown analysis of active RhoA GTPase visualized by western blot. Cells were treated with DMSO, ESE-15-one or ESE-one as above before active RhoA was precipitated. Western blot was performed on precipitated active RhoA and total cellular RhoA levels. The average of 3 independent experiments were quantified using densitometry and relative RhoA activity was calculated by dividing the active precipitated RhoA levels by the total RhoA levels. The graph shows the average with error bars representing SD. *P-value < 0.05 when compared to the DMSO control

Focal adhesion disassembly is initiated after repeated microtubule targeting and attachment events [9, 11]. Disassembling focal adhesions become signalling incompetent and no longer signal via associated kinases, Src and FAK. Therefore, we measured FAK Y397 phosphorylation as a indicator of focal adhesion signalling that can be related to its stability. FAK phosphorylation was analysed by western blot analysis. MDA-MB-231 cells were arrested in interphase with thymidine and exposed to DMSO, 0.5 μM ESE-15-one, or 0.5 μM ESE-one for 18 h after which cells were lysed and processed for western blot using antibodies targeted against phosphorylated FAK and total FAK protein (Fig. 6). Our data shows that exposure to ESE-15-one or ESE-one resulted in a four-fold increase in phosphorylated FAK. Therefore, signalling from focal adhesions is increased after exposure to either compound, suggesting that there was more FAK signalling or more focal adhesions. Along with focal adhesion signalling, actin cytoskeletal tension and fibre formation are important mediators of cell migration. These are regulated by small GTPases of which RhoA is the most important. The activity of the small Rho GTPase, RhoA was analysed by peptide pulldown assays to precipitate active RhoA. The levels of total and active RhoA were analysed by western blot. The results show that RhoA activity was significantly decreased after 18 h exposure to either compound when compared to vehicle control. ESE-15-one exposure led to a 52% decrease in RhoA activity compared to control (P < 0.004) while ESE-one exposure led to a 48% reduction in RhoA activity (P < 0.002). Together, these data suggest that there is increased signalling from the focal adhesions but a diminished level of RhoA activity which could result in poor migration.

## Discussion

Cancer cell metastasis forms an almost inevitable endpoint in tumour progression leading to complications and resistance to treatment culminating in patient death. Therefore, a large focus in cancer drug development is and should be on anti-metastatic therapies. 2-Methoxyestradiol (2ME2) is a metabolite of estrogen and was initially identified as an antiangiogenic compound [13]. Later, it was shown to also have antiproliferative effects mostly due to its ability to bind to microtubules and inhibit their dynamics leading to cell cycle arrest [13]. Clinical studies precluded further use of 2ME2 as an anticancer compound due to its lack of clinical effect as a result of its rapid metabolism and clearing [23]. As a result, a number of groups designed compounds that would retain the antiproliferative effects while withstanding clearing and metabolism inside the body. Previously, we designed and described a number of 2ME2 derivatives and their potential antiproliferative effects [19]. Design was based on the addition of a sulphamoyl moiety to the original structure along with the removal of the 3-methoxy and addition of a 2-ethyl group to make the compounds resistant to metabolic breakdown and allow reversible attachment to red blood cells via the cell surface carbonic anhydrase protein preventing clearing in the liver [19]. These sulphamoylated compounds did show antiproliferative effects in vitro in different cancer cell lines [24] although none of the non-sulphamoylated ethyl derivatives showed activity at nanomolar concentrations [20]. ESE-15-one and ESE-one are two such derivatives that have nanomolar activity in cell proliferation assays. We show here that in a triple negative metastatic cell line, both compounds reduce cell number over a 72-h period alluding to their antimitotic mechanism. This profile of cell number loss can be ascribed to their antimitotic activity which is due to an initial arrest of the cell cycle before eventual induction of apoptosis as has been shown before [25]. Thus far, all investigations on the activity of 2ME2 and the 2ME2 derivatives generated before have focused on their antiproliferative and antimitotic effect. However, little is known about their anti-metastatic potential. Since these compounds are expected to inhibit microtubule dynamics [19], we considered it possible that they may influence cell migration. Cancer metastasis depends on the change in adhesive properties of the cell to become able to adhere and migrate along mostly collagen fibres found in the stroma of the connective tissue surrounding most epithelial tissues [26]. Therefore, 2D migration as measured in vitro using wound healing assays can predict whether interventions can potentially inhibit metastasis. Microtubules have been implicated in cell migration in a number of studies. Their turnover can regulate the activity of actin fibre regulators such as GEF-H1 [27], they form the delivery channels for membrane proteins needed in focal adhesion assembly [8], and their contact with focal adhesions has been linked to the disassembly of such structures [8]. Microtubules undergo a process termed dynamic instability which means that they are continuously turned over, growing until they reach a tipping point after which they rapidly regress. Interfering in this turnover could therefore change the ability of microtubules to contact focal adhesions and would alter focal adhesion signalling and turnover leading to changes in migration. To test whether the compounds ESE-15-one and ESE-one were potential anti-migratory molecules, wound healing assays using metastatic MDA-MB-231 cells were performed and showed a significant decline in cell migration after exposure (Fig. 2). A possible confounding factor was the antimitotic effect of the compounds which meant that cells were arrested at the mitotic border where cells are rounded, poorly attached to the substrate and non-migratory. To eliminate this confounding factor, cells were first blocked in interphase through saturating concentrations of thymidine blocking the DNA synthesis process and arresting cell cycle progression in the G_1/s_ phase. When migration assays were performed with the interphase blocked cells a reduction in migration was measured in cells exposed to either ESE-15-one or ESE-one (Fig. 3). Therefore, both compounds are *bona fide* inhibitors of cancer cell migration independent of their anti-mitotic capabilities. Microtubule dynamics are vitally important for cell migration including regulating focal adhesion turnover and inducing cell polarity meaning that any impact on microtubule dynamics can have consequences for migration [8, 28]. We tested if microtubule organisation and composition was altered after compound exposure. Indeed, both ESE-15-one and ESE-one caused increases in stabilised microtubule number and length. At the same time, dynamic microtubules were disorganised with multiple centres of origin and parallel tubules found along the cell periphery instead of radiating from a single centre of origin which are the centrosomes (Fig. 5). This suggests that these compounds may inhibit cellular polarity that is dependent on the correct localisation of the centrosome. Furthermore, focal adhesion turnover is regulated in part by the microtubules. Targeting of microtubules to focal adhesions has been shown to lead to their disassembly and therefore the turnover of focal adhesions [8]. Focal adhesions signal through proteins such as focal adhesion kinase which is activated by phosphorylation [26]. Moreover, fascin-dependent microtubule stabilisation is associated with increased focal adhesion formation [29]. In addition to the abovementioned effects on the microtubules we also measured increased FAK phosphorylation and decreased RhoA GTPase activity. While the precise mechanism remains unclear we suggest that the increase in FAK activity may be due to slower turnover of focal adhesions due to a lack of microtubule mediated adhesion disassembly. RhoA GTPase activity is regulated by guanidine exchange factors. One such factor, GEF-H1, associates with the microtubules and upon release through microtubule regress activates RhoA [30]. Our data shows that the compounds inhibit RhoA activity in correlation with increased microtubule stability suggesting that through the increased binding of GEF-H1, RhoA becomes less active. This results in decreased actin fibre formation and fibre tension. Actin fibre tension is responsible for cell migration as it pulls the cell body towards the focal adhesions. Thus, a lack of tension brought about by reduced RhoA activity could also reduce cell migration.

## Conclusions

Thus, in this report we show that sulphamoylated derivatives of 2ME2, ESE-15-one and ESE-one, have bona fide antimigratory capabilities independent of their antimitotic effects. This is mediated by their ability to stabilise microtubules while inhibiting normal microtubule organisation. This is associated with increased focal adhesion signalling suggesting reduced focal adhesion turnover and decreased RhoA activity, possibly through increased microtubule association of the gef GEF-H1 leading to reduced cytoskeletal tension.

## Abbreviations

2ME2: 2-methoxyestradiol
CAIX: carbonic anhydrase IX
DAPI: 4′,6-diamidino-2-phenylindole
DMSO: dimethylsulfoxide
ECM: extracellular matrix
ESE-15-one: 2-ethyl-3-*O*-sulphamoyl-estra-1,3,5(10),15-tetraen-3-ol-17-one
ESE-one: 2-ethyl-13-methyl-17-oxo-7,8,9,11,12,13,14,15,16,17-decahydro-6-cyclopenta[*a*]phenanthrane-3 sulphamate
FAK: focal adhesion kinase
FCS: fetal calf serum
GIT: gastrointestinal tract
RT: room temperature
SDS-PAGE: sodium dodecyl sulfate polyacrylamide gel electrophoresis
