# Correction to: Novel sulphamoylated 2-methoxy estradiol derivatives inhibit breast cancer migration by disrupting microtubule turnover and organization

**DOI:** 10.1186/s12935-020-01408-3

**Published:** 2020-07-14

**Authors:** Rustelle Janse van Vuuren, Mandie Botes, Tamarin Jurgens, Anna Margaretha Joubert, Iman van den Bout

**Affiliations:** 1 Department of Physiology, University of Pretoria, Pretoria, 0084 South Africa; 2 Centre for Neuroendocrinology, University of Pretoria, Pretoria, 0084 South Africa

## Correction to: Cancer Cell Int (2019) 19:1 10.1186/s12935-018-0719-4

Following publication of the original article [1], the authors notified us that the graph presented in Figure 3a is the same as Figure 2a in the published manuscript. Figure 3 below represents the true migration values achieved for cells blocked in interphase and treated with the different compounds.

**Fig. 3:**
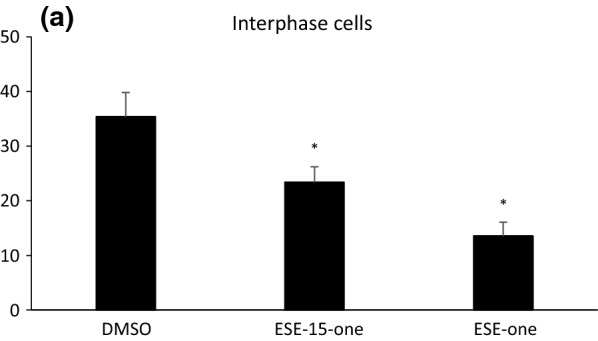
Migration of interphase arrested cells is inhibited by ESE-15-one and ESE-one exposure. **a** MDA-MB-231 cells were first blocked in G1/S by exposure to thymidine before cell free zones were generated and cells were exposed to 0.2% DMSO, 0.5 μM ESE-15-one or 0.5 μM ESE-one. Cell migration into the cell-free zone was quantified after 18 h. The graph represents the average of at least three repeats with error bars representing SEM. *P < 0.001 in t-test comparison with DMSO-treated cells. **b** Light microscopy images of cell migration assays showing interphase cells at time 0 h and after 18 h treated with DMSO, ESE-15-one or ESE-one. Scale bar is 400 μm

Specifically, blocked cells treated with DMSO closed 35% of the wound while ESE-15-one reduced that to 23% and ESE one reduced this to 13%. T tests show statistical significance.
